# Supplementary material for: Vitamin D Modified DSS-Induced Colitis in Mice via STING Signaling Pathway
Source: Biology (Basel). 2025 Jun 18;14(6):715. doi: 10.3390/biology14060715 (PMC12190092; doi:10.3390/biology14060715)
Supplement: Supplementary file 1 [file biology-14-00715-s001.zip › Supplementary Materials S1: 16s-report of gut microbiota/Groups/diff_analysis/heatmap/METACYCdiffheatmap.pdf]

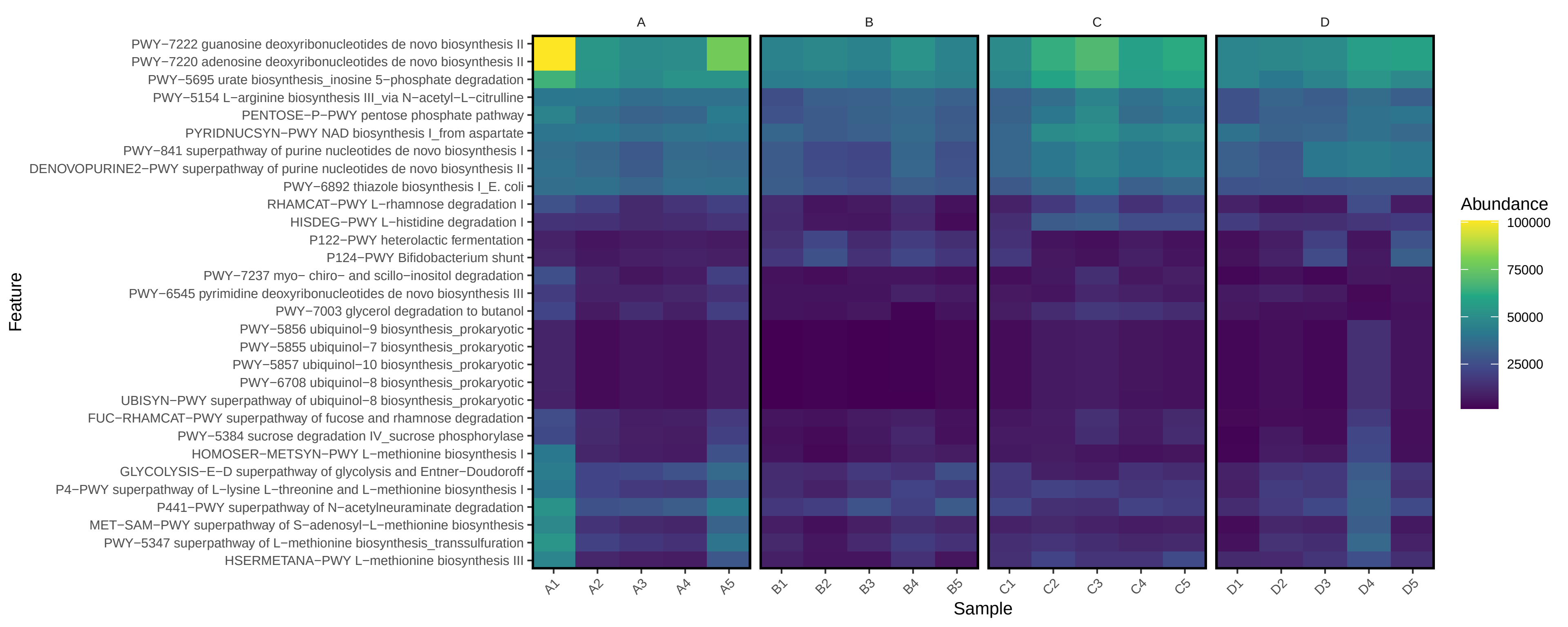

A
B
C
D
PWY−7222 guanosine deoxyribonucleotides de novo biosynthesis II
PWY−7220 adenosine deoxyribonucleotides de novo biosynthesis II
PWY−5695 urate biosynthesis_inosine 5−phosphate degradation
PWY−5154 L−arginine biosynthesis III_via N−acetyl−L−citrulline
PENTOSE−P−PWY pentose phosphate pathway
PYRIDNUCSYN−PWY NAD biosynthesis I_from aspartate
PWY−841 superpathway of purine nucleotides de novo biosynthesis I
DENOVOPURINE2−PWY superpathway of purine nucleotides de novo biosynthesis II
PWY−6892 thiazole biosynthesis I_E. coli
RHAMCAT−PWY L−rhamnose degradation I
HISDEG−PWY L−histidine degradation I
P122−PWY heterolactic fermentation
P124−PWY Bifidobacterium shunt
PWY−7237 myo− chiro− and scillo−inositol degradation
PWY−6545 pyrimidine deoxyribonucleotides de novo biosynthesis III
PWY−7003 glycerol degradation to butanol
PWY−5856 ubiquinol−9 biosynthesis_prokaryotic
PWY−5855 ubiquinol−7 biosynthesis_prokaryotic
PWY−5857 ubiquinol−10 biosynthesis_prokaryotic
PWY−6708 ubiquinol−8 biosynthesis_prokaryotic
UBISYN−PWY superpathway of ubiquinol−8 biosynthesis_prokaryotic
FUC−RHAMCAT−PWY superpathway of fucose and rhamnose degradation
PWY−5384 sucrose degradation IV_sucrose phosphorylase
HOMOSER−METSYN−PWY L−methionine biosynthesis I
GLYCOLYSIS−E−D superpathway of glycolysis and Entner−Doudoroff
P4−PWY superpathway of L−lysine L−threonine and L−methionine biosynthesis I
P441−PWY superpathway of N−acetylneuraminate degradation
MET−SAM−PWY superpathway of S−adenosyl−L−methionine biosynthesis
PWY−5347 superpathway of L−methionine biosynthesis_transsulfuration
HSERMETANA−PWY L−methionine biosynthesis III
Feature
A1
A2
A3
A4
A5
B1
B2
B3
B4
B5
C1
C2
C3
C4
C5
D1
D2
D3
D4
D5
Sample
Abundance
100000
75000
50000
25000
